# Menorrhagia, Etc., and Hygienics

**Published:** 1888-06

**Authors:** John Meredith

**Affiliations:** Medical Officer of Health, Wellington, Somerset


### MENORRHAGIA, ETC., AND HYGIENICS.

BY

JOHN MEREDITH, M.D., Edin.,

*Medical Officer of Health, Wellington, Somerset.*

(Continued from p. 27.)

---

THE fact of Mrs. R. F.'s metrorrhagia being first brought on by a strain leads me to mention another case which was aggravated in that way.

I have already published the details of this case—*British Medical Journal*, 8th Nov., 1879,—and I only wish to recapitulate some of them here with the view of pointing out some features in the history of the case which have been revealed by the light of subsequent events. The outline of the case is as follows:—E. F., a young woman, aged 20, daughter of healthy parents, suffered from excessive menstrual discharge. One day in March of 1877, while she was serving as a housemaid with a family at Weston, she exerted herself in lifting a bookcase, and she was menstruating at the time. She felt the strain affecting her, and the discharge, instead of terminating at the expected time, continued to flow day after day, and in consequence she had to give up her situation. I am not in a position to say anything about the sanitary state of the house at Weston. When I saw her at her own home some little time afterwards, she seemed exhausted from loss of blood and suffering from pain in the hypogastric region.

Various means were resorted to for the purpose of arresting the hæmorrhage: among them, subcutaneous injection of ergotine, application of acid carbolic to the uterus internally, at another time of nitrate of silver—and now and again with success, at all events for the time. It is not necessary here to repeat all that was done, beyond saying that I never had a case of the kind which occupied more of my thoughts and attention. After doing all I could for the case, and finding that in spite of all the loss persisted, I desired the patient to go to Birmingham, to be under the care of Mr. Lawson Tait; accordingly, towards the end of January, 1878, she went there. Mr. Tait was not able, any more than I was, to find any organic lesion. The fundus of the uterus was a little enlarged, nothing else—no displacement of any kind.

The patient, after undergoing a few local applications to the interior of the womb, rapidly recovered, and was able to leave the Women's Hospital for a Convalescent Home on the 1st of March. After staying for a while here she returned to her home, to all appearances well. But on the very day of her return, and a few hours after her arrival, the menorrhagia returned, and persisted as it had done before, notwithstanding every effort made to arrest it; and the patient's condition was becoming critical in every sense of the word. Seeing this, I again advised her to return to Birmingham and have the uterine appendages removed, if no other means of saving her presented itself. I had been in correspondence with Mr. Tait about the case. Accordingly, on the 5th of August she was taken to Birmingham again to be under Mr. Tait's care; and on the 8th he removed both ovaries. The patient made an excellent recovery in every respect, and in due course returned to her friends. She has since been living in domestic service and in the enjoyment of good health.

As to the condition of the ovaries, when removed they were found to be "enlarged and flabby, with traces of lymph in their neighbourhood." No other morbid appearance. That is the outline of the case, and the question which further experience and observations suggest is—Was it after all absolutely necessary to remove those appendages? At the time I thought it was; and I must take the chief responsibility for advising the patient to consent to the operation. I think now, however, that the operation should, at least, have been deferred until we should have seen what change of air, &c., would have done—until one could have seen what the effect of residence in a healthy dwelling with a supply of pure water could do. Judging from what I believe to have taken place in a case which had a great deal in common with the one under notice, the consequence probably would be an arrest of the unnatural flow and return to a state of ordinary health.

The condition of the ovaries when removed showed only that they had had an extra amount of work to do, and that of an abnormal character, and had, like any other glands, increased in size to meet the extra demand.

Psychologists at times consider it imprudent to send patients, who may have to all appearances, recovered from certain mental disorders back to their old homes, with their familiar surroundings and influences, immediately after discharge from special care; since such associations are apt to bring about relapses. There is not much difference, it seems to me, between psychical and physical influences in this connection.

The main offending element in this case was, in my opinion, the pump-water. The uncongenial agent contained in this, finding access to the ovaries through the blood, re-kindled the slumbering susceptibility by invading the glands before they had had time to assert the independence of assured health.

I must add that at the time I was attending upon this patient I had not realised that impure water was capable of playing an all-important part in the functional economy of certain people. Further: upon making inquiries subsequently, I learnt that others, who were using this pump-water, suffered; some in like manner to this patient, the rest differently; but who did not so suffer when they had good potable water to drink.

On the 17th of July, 1883, I called to see E. F.'s father, an old man suffering from catarrhal affections, and I had a long chat with her mother at the same time. The old lady was not well either. She complained of giddiness, gastric pains, and sudden fits of epistaxis. She had had these bleedings from the nose frequently of late, and she complained of the languor so generally associated with these losses.

Enquiring into the condition of things surrounding her, I learnt that the water which she and her family had for drinking and cooking was not good. The best description of it was to call it ditch-water, for it was procured from the side of a hedge. Some months before, when she was able to get good water for potable purposes, her health was good, and she had no bleedings from the nose.

When the daughter returned to her home when first taken with menorrhagia, the only water obtainable was this water procured from the side of a hedge in a field. The family removed to another house during her illness, which had a well with a pump fixed in it, but this well was open to pollution from soakage from a garden-plot close to, besides infiltration from a defective drain.

I adduce the mother's case to show that there is apparently a family liability to hæmorrhage.

It is often said that the climate of this Vale of Taunton Dean is "relaxing," and therefore predisposes persons to hæmorrhages of one form or another. The only explanation which I have been able to give of this alleged condition is, that the depressing cause is not necessarily in the air of the district, but rather in immediate relationship with the abodes of the persons suffering from the "relaxed" feeling. Here is a case in point, and I might give many more to the same effect.

Miss H., aged 34, informed me some years ago that when she lived in the North of England, she always felt so well. The menses recurred with regularity, and were not excessive in any way; but that as soon as she came home to her native village where her mother resided the courses became excessive, double what they should be, and lasting a week or more each time, and returning every three weeks or earlier.

Disturbance of the catamenia was one of the first discomforts she experienced after returning home. Next came hæmorrhoids, with gastro-hepatic disorders.

There was nothing in the surroundings of Miss H.'s home that could be found fault with, except that the water used was not, in my opinion, wholesome and safe. It was obtained from a well polluted by surface-drainage—in this way: the well was in the back kitchen, provided with a pump; the pump-trough stood partly over the mouth of the well, and the escape from the trough was on rough paving-stones uncemented, with an outlet through the wall out into the orchard, and thence slowly away from the house.

One need hardly say that the pump-trough is the common receptacle of various washings, &c., and it was manifest that a certain proportion of the liquids soaked, through the pavement and the porous earth under, back into the well. This form of pump-arrangement used to be common in this district at one time, and it is not by any means wholly done away with yet.

Some time ago Miss H.'s mother died, and she left the village for another place of residence where the water supply was good, and the result has been in every way beneficial to her health. There is nothing relaxing about the climate now. In regard to the old house, the new tenant has had the defects about the mouth of the well put to rights, and that with advantage to the health of his family.

In cases of women who are peculiarly liable to hæmorrhages, those in whom it is the weak link, as it were, in the constitutional chain, in such persons almost any noxious element may prolong a menstrual course, or aggravate the flow, or, it may be, induce it out of time. I am persuaded that instances of these are far from being uncommon.

In one case a severe aggravation of the flow resulted from exposure to a leaky gas-pipe; and defective gas-taps are responsible, in my opinion, for much more suffering than is commonly imagined. So are imperfect water-closets and drains.

I have mentioned one case where I considered that impure water was the cause of epistaxis and the "out-of-sorts" feeling which the patient complained of at the same time. I have met with many such cases, and in each the inquiries made into the surroundings as affecting health showed that there was some serious unhealthy influence or other at work. Take one as example—this time from among the male sex.

J. H., aged 18, clerk in a warehouse, came to me in September, 1883, complaining of being weak; had lost weight lately, and suffered from frequent attacks of epistaxis; said he felt languid, and suffered from headache; was thirsty, and drank a good deal of water. The fauces were red.

He admitted that he had been exposed to bad smells—drains chiefly. He was a total abstainer from alcohol, and the water which he so freely partook of was of a suspicious quality, as it was obtained from a well liable to pollution from surface-soakage. By way of treatment, I directed him to keep away from the unhealthy odours he had mentioned; live out of doors in the open air as much as he could; and, as the water was not of a desirable nature, to drink a glass of porter with his meals once or twice a day; and, further, to take ten grains of chlorate of potash with iron thrice daily. He went away and returned in about a week, saying he was much better; had had a little bleeding once, and in a few days more he came and expressed himself as feeling all right.

In regard to the epistaxis in this case, resulting as I take it from vitiation of the blood, the Schneiderian membrane but played the part of a safety-valve to the surcharged vascular system—a gland striving to excrete foreign matter. But it was a morbid action: since the quality of the coats of blood-vessels—that is, their power to carry and retain their contents—depends upon the quality of the blood circulating in them, when this is diseased their efficiency is lessened.

In all cases of regularly recurring epistaxis, or in those where the regularity is not immediately obvious, I have found that the sufferers were exposed to some insanitary influence or other, if not to a combination of such things.

I have notes of cases of so-called vicarious catamenia. Most of them refer to girls of 16 years old and upwards. Some suffered from more or less copious bleedings from the nose every month regularly; in others, regular periodicity did not obtain.

This sort of thing occurred in two cases for several years before the natural course asserted itself. Now as to the hygienic conditions under which these persons lived: Every one of them lived in dwellings of an unhealthy sort, or were exposed to some form of nuisance, *e.g.* drain-smells, impure water, &c. Then there were instances where the domestic habits of the family had to be taken into account, such as disregard of proper ventilation of the house and approved cleanliness.

I have often been struck with certain traits of heredity that prevail at times under these circumstances. As the mother's house is ordered, so is the daughter's, and the history, I suspect, might be carried on further still. The houses possess a sort of family smell in them, which to an outsider may be pungently perceptible, and objectionable as well; but which the family are unconscious of. Nevertheless, members of such families do not escape from the consequences of these influences; some one or other of them is often ailing, and none of them are in a bracing state of good health.

To continue my illustrations. I will briefly sketch the case of Mrs. A. It is similar to others. I made a note of the case in March, 1886. The patient was a strong woman, 40 years of age, a factory labourer. She came to Somerset from Yorkshire in the summer of 1884. Since her arrival her catamenia had been excessive, a new experience altogether for her. A period lasted a couple of weeks or more. Her house was in the country; it seemed damp, as the floor was below the level of the road. It had some malodorous matters near the door, which she averred were due to a neighbour woman and not to herself. The water used by her and her neighbours was obtained from a well liable to pollution from surface-water.

I advised my patient to have the nuisance removed from the neighbourhood of her doorway, and have the mouth of the well better guarded, and be cleanly in other respects as well. In the way of medicines, she had some tincture of cannabis Ind., chlorate of potash with glycerine. The recommendations were acted upon, and in due course the patient's health improved, and the menses resumed their normal character.

Mrs. A.'s neighbour deserves a passing remark besides that of causing a nuisance to others living near her. This neighbour, M. F., a widow about 55 years old now, suffered from menorrhagia, with but brief intervals, for some twelve years. The flow stopped with her when she was about 53. She was under my care, off and on, during a good part of the time of her suffering. There was no tumour in the uterus, but its fundus was retroverted.

During her excessive catamenial period, while she was under my care I used every form of treatment that I could think of as likely to do good in the case, but nothing appeared to produce any lasting benefit. I had not fully realised when attending poor M. F. that hygienic conditions had so much to do as I am now persuaded they have with ailments such as hers.

During the last year or two prior to the cessation, and occasionally before that, she was under the care of other medical practitioners; and it may, in fact, be said of her, as was said of another afflicted in a similar way something like nineteen centuries ago, "that she suffered much from many physicians." Nevertheless, however fitting the indictment, it seems to me nothing but natural to infer that she yet suffered much more from her own insanitary ways and unhealthy dwelling.

M. F. is now very much of a despondent invalid, like one whose blood-making function has been undergoing so protracted an overstrain that it has not power left to resume the normal tale of healthful action. Her daughter living at home has much of her mother's domestic ways, and suffered, when I made my report, from excess at her monthly periods. It seems to be an accepted fact among us, that "the causes of these climacteric hæmorrhages are really not known." I make this quotation from Quain's *Dictionary of Medicine*. M. F.'s case was a typical one. Every general practitioner must be familiar with many cases of the kind. In every instance where I have investigated into the condition of matters surrounding them, I have found some unhealthy influence or other at work. It may at present be too much to say that mal-hygiene is the actual cause of these hæmorrhages; but it is at least a potential one.

I would observe next that menstrual disorders of the kind I have been describing seem more prevalent in the springtime of the year, or are more accentuated in character then, than during other periods. Whether this may be due to the barometric and thermometric changes incidental to that season, I am not prepared to say; or whether *bacilli*, changing character with altered environment; or the prolific microbes of stagnant water; or yet some other form of the bacterial entity, which is credited with entering into all forms of zymotic action, acquires an activity then, circumstances being favourable, beyond what it possesses at other times, can but be a matter of conjecture.

All this, however, is a field open to imagination, and the just exercise of imagination is as much called for in medicine as it is in physics.

Further, in common with medical practitioners following the profession in India and other places in the Tropics, I had to deal with cases of excessive menstruation, and I well remember the anxiety which I often felt concerning them. Instead of trusting so much to drugs and local applications, &c., it would be better to see to the quality of the water used; guard against pollution of the supply by surface-currents and the like, and of course against every element of an unhealthy nature.

The most serious cases of *post partum* hæmorrhage which I can call to mind were those which occurred in unhealthy houses, where the water-supply and the drainage were bad.

